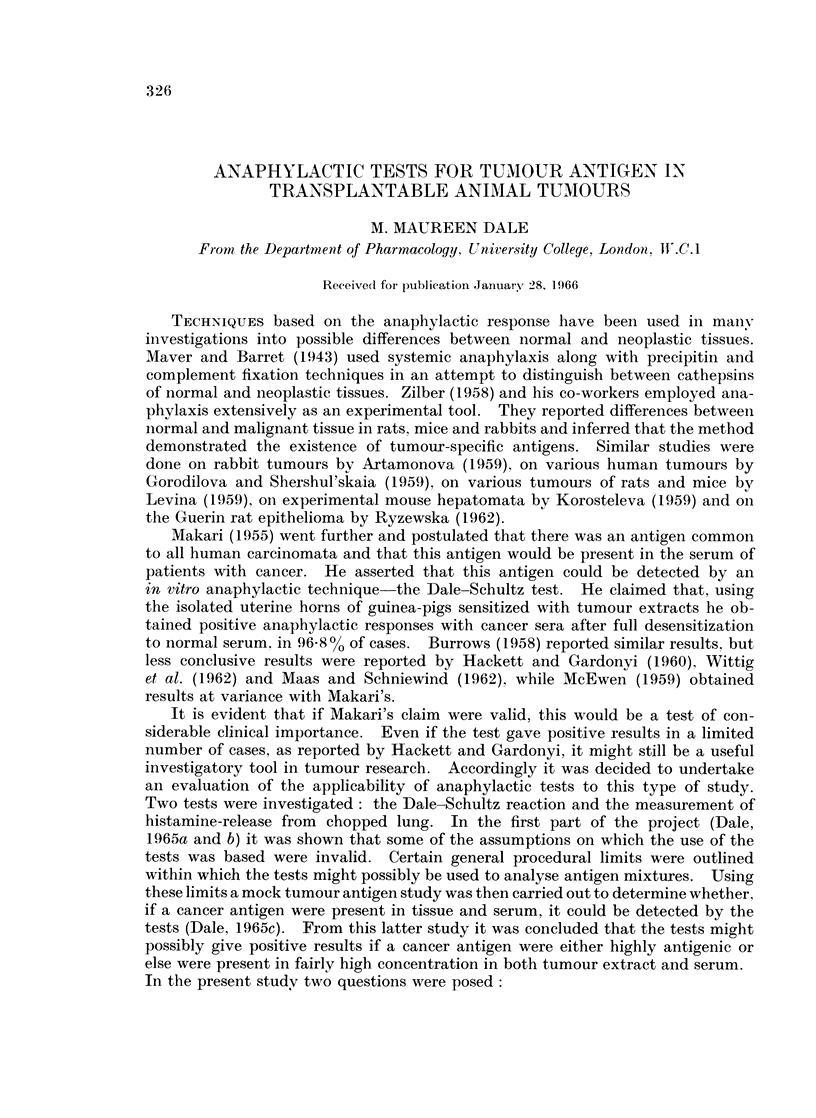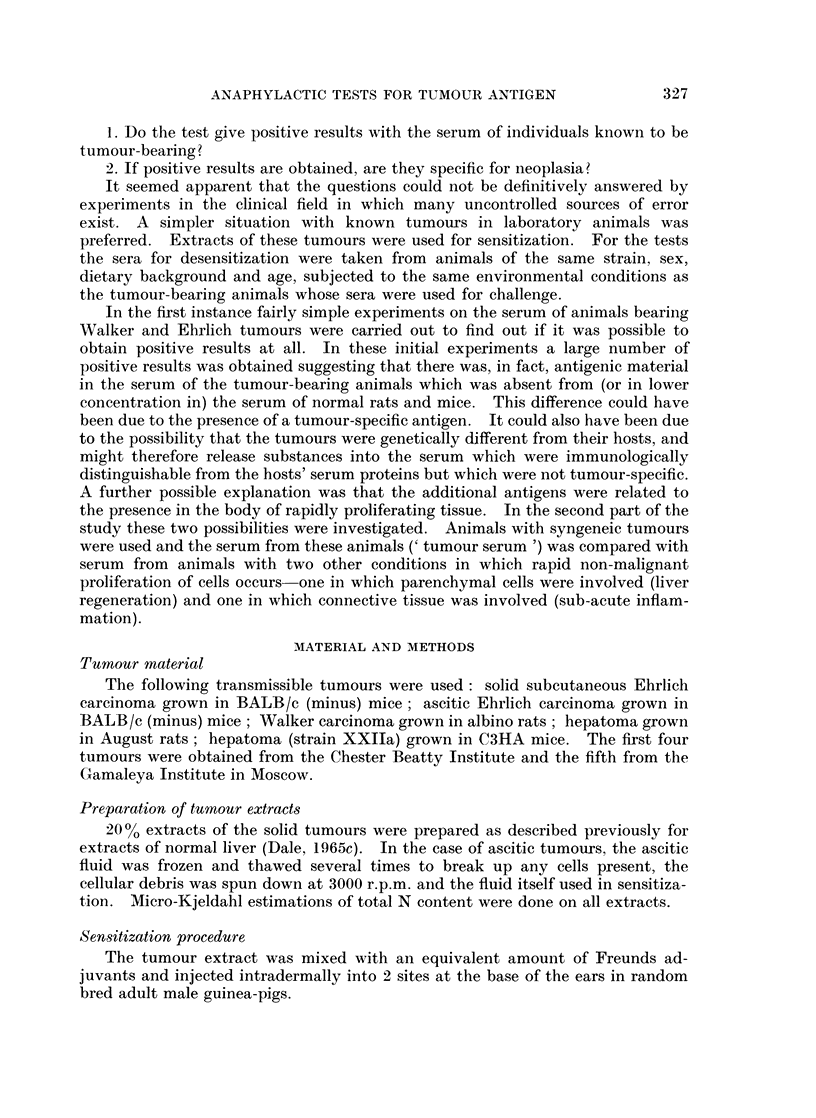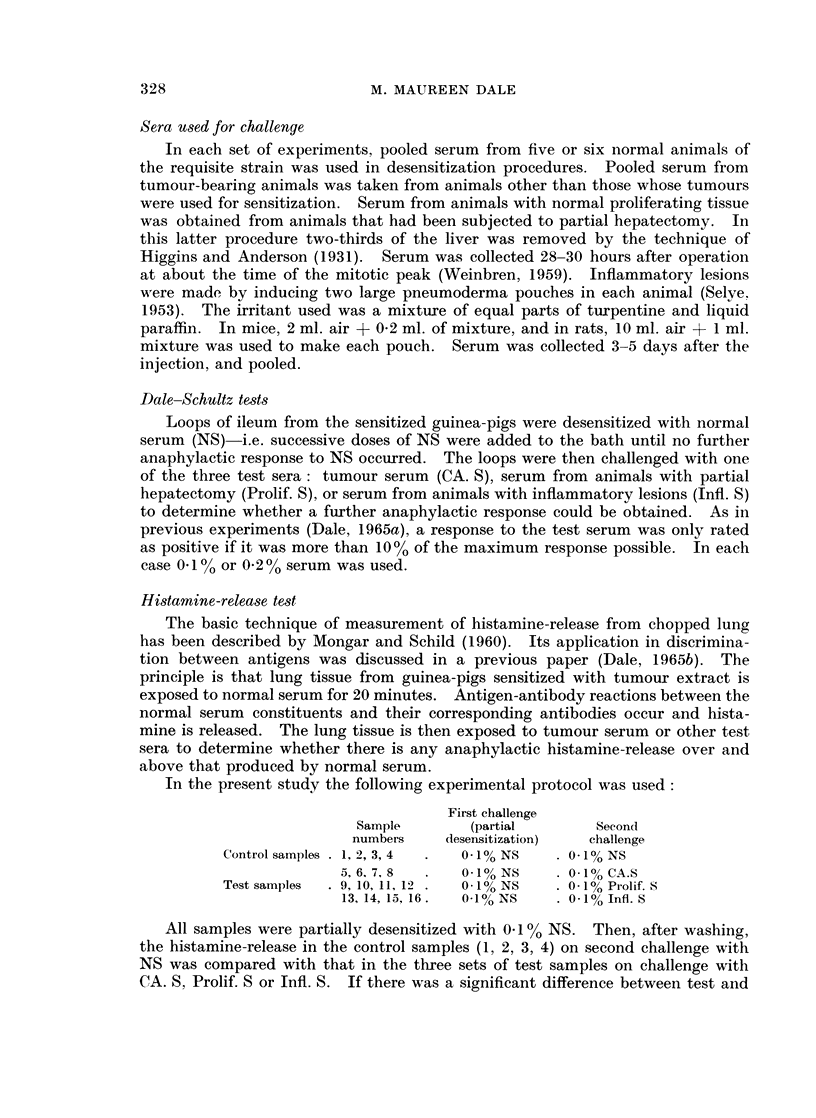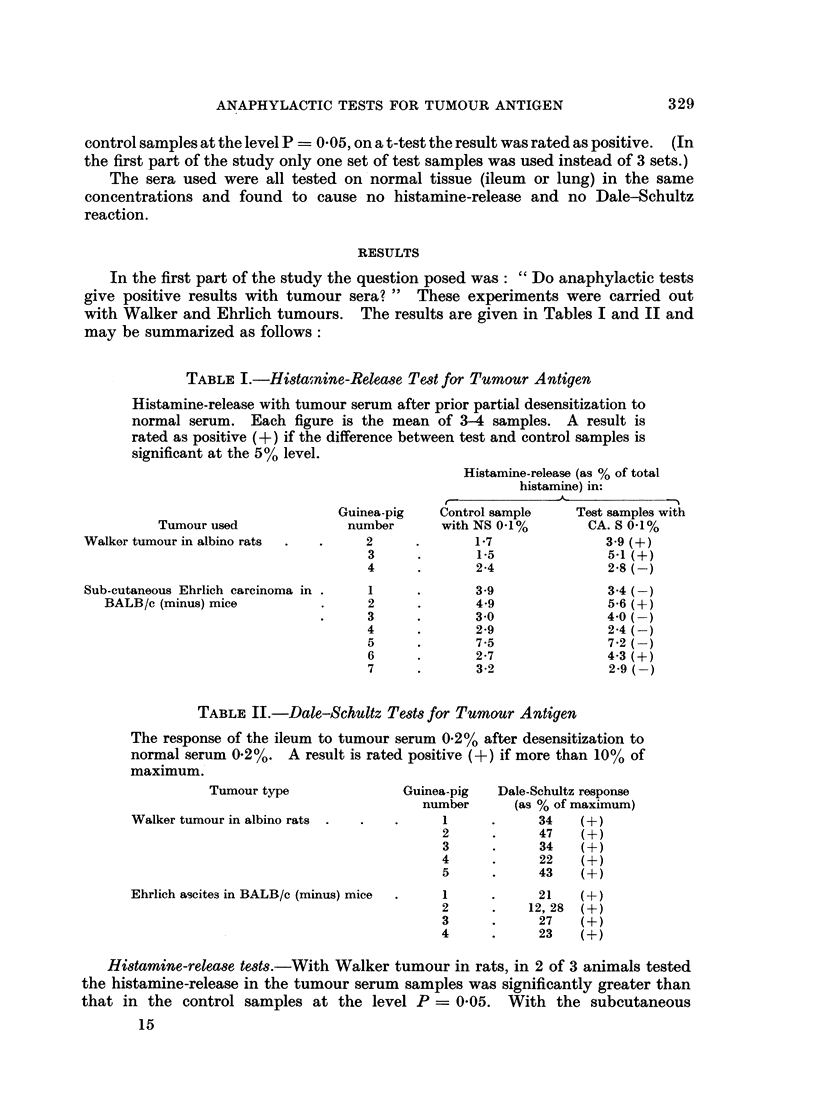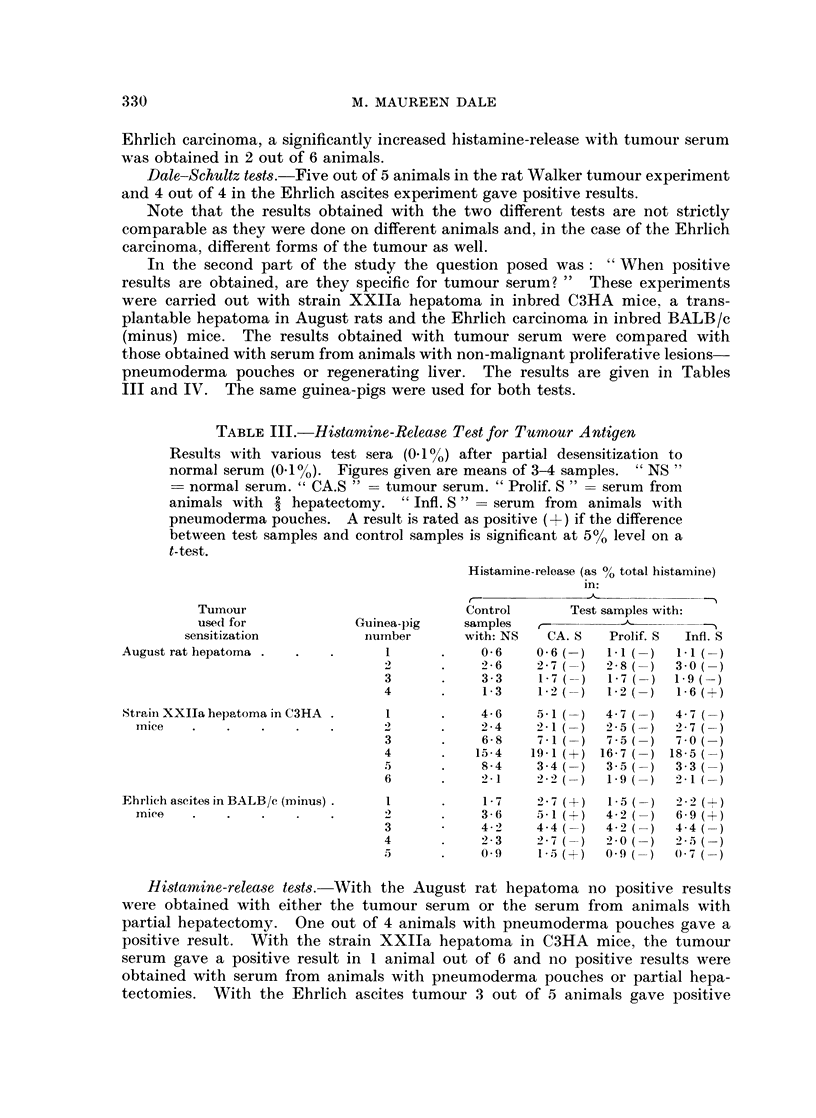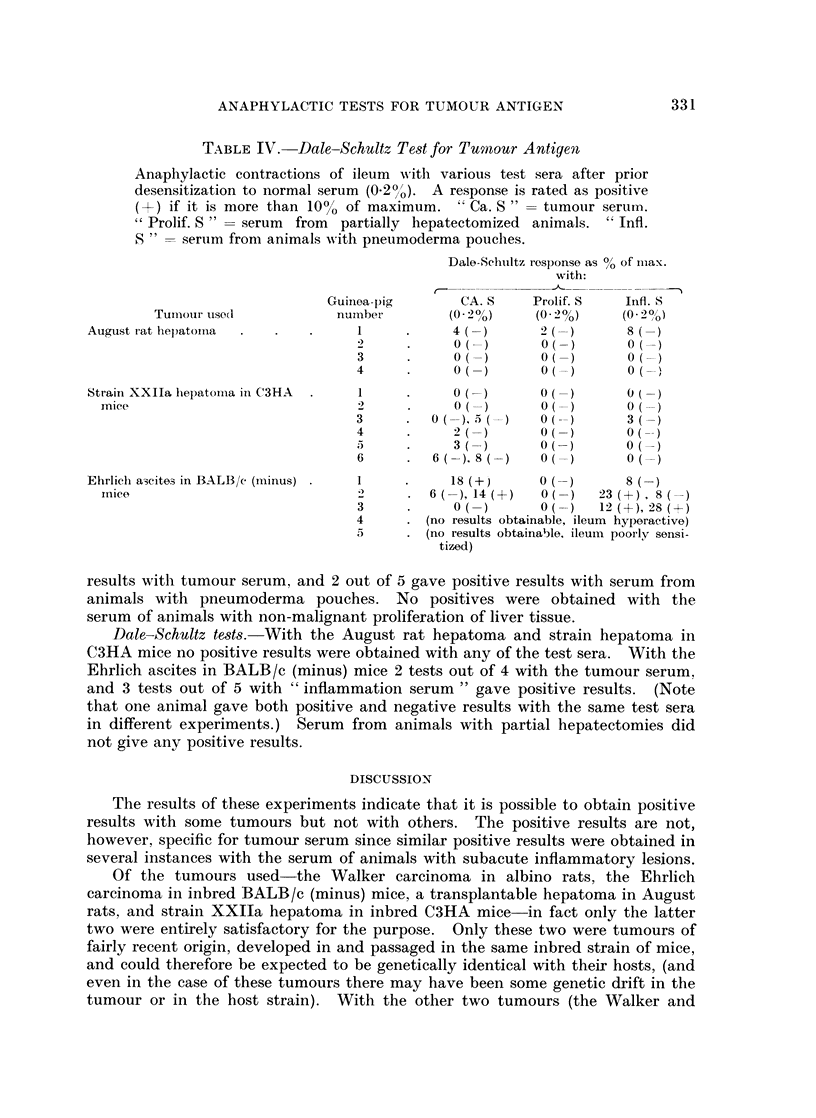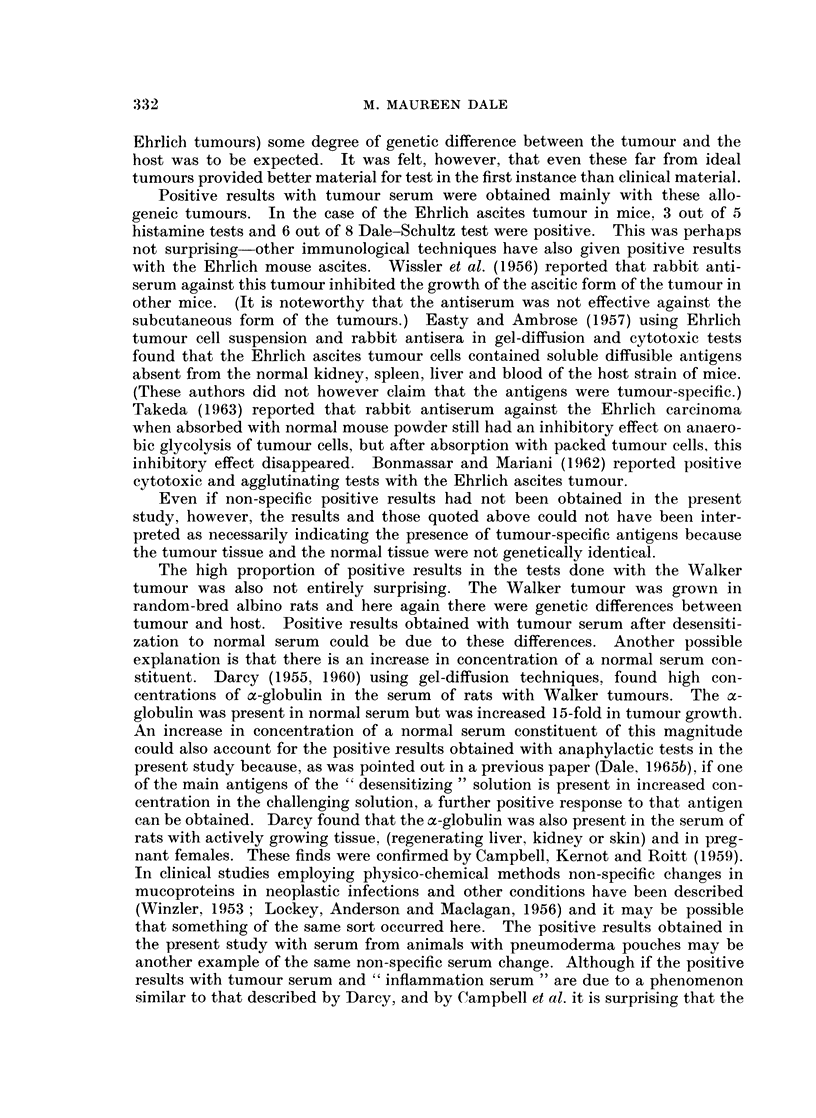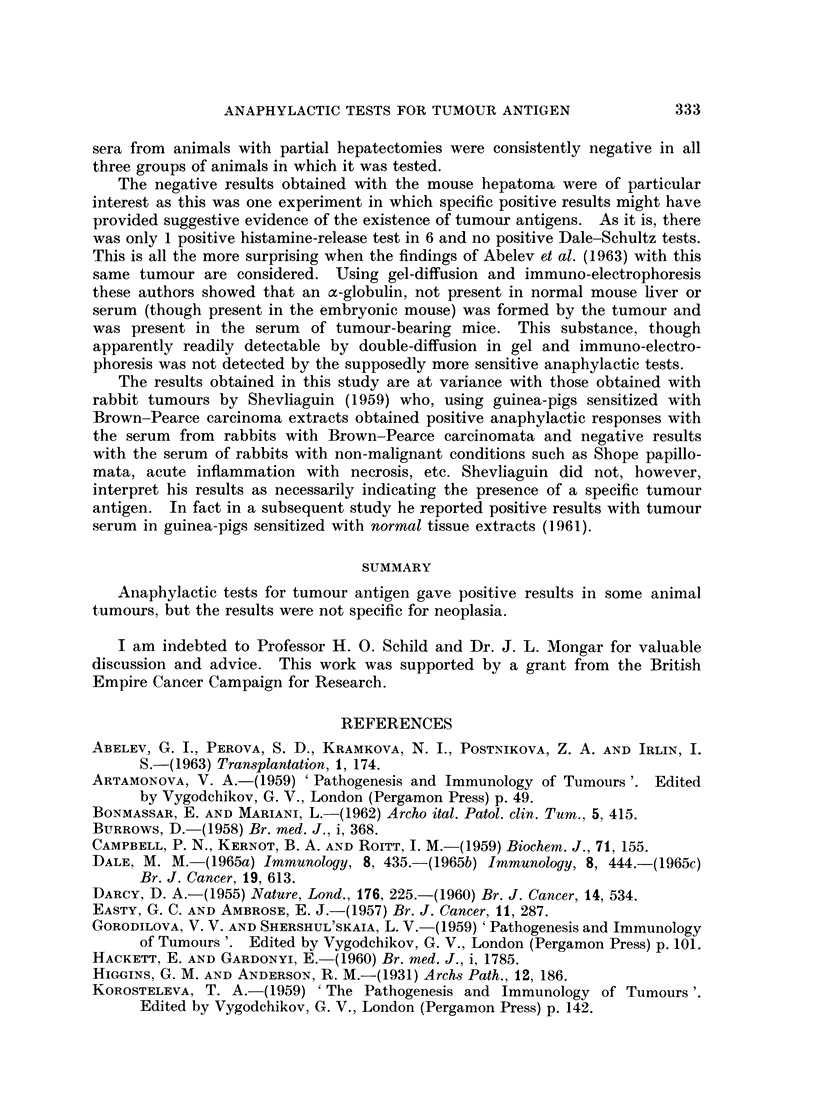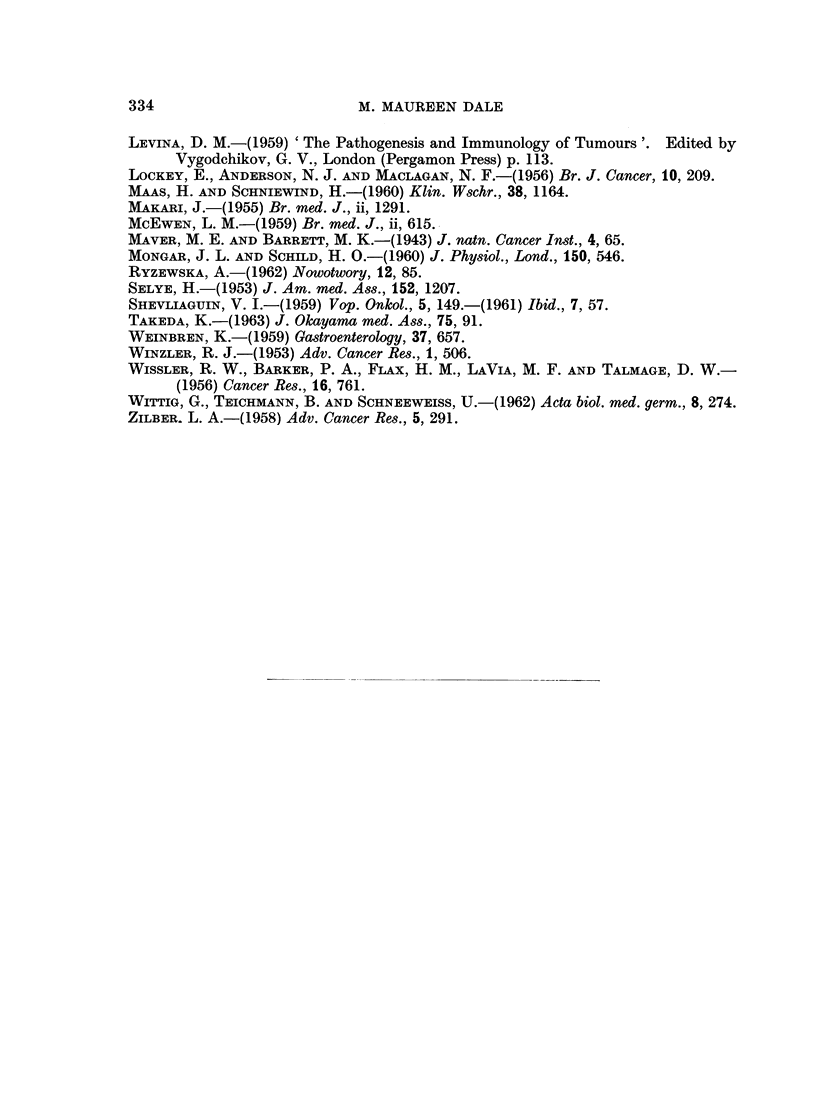# Anaphylactic tests for tumour antigen in transplantable animal tumours.

**DOI:** 10.1038/bjc.1966.40

**Published:** 1966-06

**Authors:** M. M. Dale


### ANAPHYLACTIC TESTS FOR TUMOUR ANTIGEN IN TRANSPLANTABLE ANIMAL TUMOURS

M. MAUREEN DALE

*From the Department of Pharmacology, University College, London, W.C.1*

Received for publication January 28, 1966

TECHNIQUES based on the anaphylactic response have been used in many investigations into possible differences between normal and neoplastic tissues. Maver and Barret (1943) used systemic anaphylaxis along with precipitin and complement fixation techniques in an attempt to distinguish between cathepsins of normal and neoplastic tissues. Zilber (1958) and his co-workers employed anaphylaxis extensively as an experimental tool. They reported differences between normal and malignant tissue in rats, mice and rabbits and inferred that the method demonstrated the existence of tumour-specific antigens. Similar studies were done on rabbit tumours by Artamonova (1959), on various human tumours by Gorodilova and Shershul'skaia (1959), on various tumours of rats and mice by Levina (1959), on experimental mouse hepatomata by Korosteleva (1959) and on the Guerin rat epithelioma by Ryzewska (1962).

Makari (1955) went further and postulated that there was an antigen common to all human carcinomata and that this antigen would be present in the serum of patients with cancer. He asserted that this antigen could be detected by an *in vitro* anaphylactic technique—the Dale–Schultz test. He claimed that, using the isolated uterine horns of guinea-pigs sensitized with tumour extracts he obtained positive anaphylactic responses with cancer sera after full desensitization to normal serum, in 96·8% of cases. Burrows (1958) reported similar results, but less conclusive results were reported by Hackett and Gardonyi (1960), Wittig *et al.* (1962) and Maas and Schniewind (1962), while McEwen (1959) obtained results at variance with Makari's.

It is evident that if Makari's claim were valid, this would be a test of considerable clinical importance. Even if the test gave positive results in a limited number of cases, as reported by Hackett and Gardonyi, it might still be a useful investigatory tool in tumour research. Accordingly it was decided to undertake an evaluation of the applicability of anaphylactic tests to this type of study. Two tests were investigated : the Dale–Schultz reaction and the measurement of histamine-release from chopped lung. In the first part of the project (Dale, 1965*a* and *b*) it was shown that some of the assumptions on which the use of the tests was based were invalid. Certain general procedural limits were outlined within which the tests might possibly be used to analyse antigen mixtures. Using these limits a mock tumour antigen study was then carried out to determine whether, if a cancer antigen were present in tissue and serum, it could be detected by the tests (Dale, 1965*c*). From this latter study it was concluded that the tests might possibly give positive results if a cancer antigen were either highly antigenic or else were present in fairly high concentration in both tumour extract and serum. In the present study two questions were posed :

1. Do the test give positive results with the serum of individuals known to be tumour-bearing?

2. If positive results are obtained, are they specific for neoplasia?

It seemed apparent that the questions could not be definitively answered by experiments in the clinical field in which many uncontrolled sources of error exist. A simpler situation with known tumours in laboratory animals was preferred. Extracts of these tumours were used for sensitization. For the tests the sera for desensitization were taken from animals of the same strain, sex, dietary background and age, subjected to the same environmental conditions as the tumour-bearing animals whose sera were used for challenge.

In the first instance fairly simple experiments on the serum of animals bearing Walker and Ehrlich tumours were carried out to find out if it was possible to obtain positive results at all. In these initial experiments a large number of positive results was obtained suggesting that there was, in fact, antigenic material in the serum of the tumour-bearing animals which was absent from (or in lower concentration in) the serum of normal rats and mice. This difference could have been due to the presence of a tumour-specific antigen. It could also have been due to the possibility that the tumours were genetically different from their hosts, and might therefore release substances into the serum which were immunologically distinguishable from the hosts' serum proteins but which were not tumour-specific. A further possible explanation was that the additional antigens were related to the presence in the body of rapidly proliferating tissue. In the second part of the study these two possibilities were investigated. Animals with syngeneic tumours were used and the serum from these animals (' tumour serum ') was compared with serum from animals with two other conditions in which rapid non-malignant proliferation of cells occurs—one in which parenchymal cells were involved (liver regeneration) and one in which connective tissue was involved (sub-acute inflammation).

### MATERIAL AND METHODS

### *Tumour material*

The following transmissible tumours were used : solid subcutaneous Ehrlich carcinoma grown in BALB/c (minus) mice ; ascitic Ehrlich carcinoma grown in BALB/c (minus) mice ; Walker carcinoma grown in albino rats ; hepatoma grown in August rats ; hepatoma (strain XXIIa) grown in C3HA mice. The first four tumours were obtained from the Chester Beatty Institute and the fifth from the Gamaleya Institute in Moscow.

### *Preparation of tumour extracts*

20% extracts of the solid tumours were prepared as described previously for extracts of normal liver (Dale, 1965*c*). In the case of ascitic tumours, the ascitic fluid was frozen and thawed several times to break up any cells present, the cellular debris was spun down at 3000 r.p.m. and the fluid itself used in sensitization. Micro-Kjeldahl estimations of total N content were done on all extracts.

### *Sensitization procedure*

The tumour extract was mixed with an equivalent amount of Freunds adjuvants and injected intradermally into 2 sites at the base of the ears in random bred adult male guinea-pigs.

*Sera used for challenge*

In each set of experiments, pooled serum from five or six normal animals of the requisite strain was used in desensitization procedures. Pooled serum from tumour-bearing animals was taken from animals other than those whose tumours were used for sensitization. Serum from animals with normal proliferating tissue was obtained from animals that had been subjected to partial hepatectomy. In this latter procedure two-thirds of the liver was removed by the technique of Higgins and Anderson (1931). Serum was collected 28–30 hours after operation at about the time of the mitotic peak (Weinbren, 1959). Inflammatory lesions were made by inducing two large pneumoderma pouches in each animal (Selye, 1953). The irritant used was a mixture of equal parts of turpentine and liquid paraffin. In mice, 2 ml. air + 0·2 ml. of mixture, and in rats, 10 ml. air + 1 ml. mixture was used to make each pouch. Serum was collected 3–5 days after the injection, and pooled.

*Dale–Schultz tests*

Loops of ileum from the sensitized guinea-pigs were desensitized with normal serum (NS)—i.e. successive doses of NS were added to the bath until no further anaphylactic response to NS occurred. The loops were then challenged with one of the three test sera : tumour serum (CA. S), serum from animals with partial hepatectomy (Prolif. S), or serum from animals with inflammatory lesions (Infl. S) to determine whether a further anaphylactic response could be obtained. As in previous experiments (Dale, 1965*a*), a response to the test serum was only rated as positive if it was more than 10% of the maximum response possible. In each case 0·1% or 0·2% serum was used.

*Histamine-release test*

The basic technique of measurement of histamine-release from chopped lung has been described by Mongar and Schild (1960). Its application in discrimination between antigens was discussed in a previous paper (Dale, 1965*b*). The principle is that lung tissue from guinea-pigs sensitized with tumour extract is exposed to normal serum for 20 minutes. Antigen-antibody reactions between the normal serum constituents and their corresponding antibodies occur and histamine is released. The lung tissue is then exposed to tumour serum or other test sera to determine whether there is any anaphylactic histamine-release over and above that produced by normal serum.

In the present study the following experimental protocol was used :

| | Sample numbers | First challenge (partial desensitization) | Second challenge |
|---|---|---|---|
| Control samples . | 1, 2, 3, 4 . | 0·1% NS | . 0·1% NS |
| | 5, 6, 7, 8 . | 0·1% NS | . 0·1% CA.S |
| Test samples . | 9, 10, 11, 12 . | 0·1% NS | . 0·1% Prolif. S |
| | 13, 14, 15, 16 . | 0·1% NS | . 0·1% Infl. S |

All samples were partially desensitized with 0·1% NS. Then, after washing, the histamine-release in the control samples (1, 2, 3, 4) on second challenge with NS was compared with that in the three sets of test samples on challenge with CA. S, Prolif. S or Infl. S. If there was a significant difference between test and control samples at the level P = 0·05, on a t-test the result was rated as positive. (In the first part of the study only one set of test samples was used instead of 3 sets.)

The sera used were all tested on normal tissue (ileum or lung) in the same concentrations and found to cause no histamine-release and no Dale–Schultz reaction.

### RESULTS

In the first part of the study the question posed was : " Do anaphylactic tests give positive results with tumour sera? " These experiments were carried out with Walker and Ehrlich tumours. The results are given in Tables I and II and may be summarized as follows :

TABLE I.—*Histamine-Release Test for Tumour Antigen*

Histamine-release with tumour serum after prior partial desensitization to normal serum. Each figure is the mean of 3–4 samples. A result is rated as positive (+) if the difference between test and control samples is significant at the 5% level.

| Tumour used | Guinea-pig number | Histamine-release (as % of total histamine) in: Control sample with NS 0·1% | Test samples with CA. S 0·1% |
|---|---|---|---|
| Walker tumour in albino rats | 2 | 1·7 | 3·9 (+) |
| | 3 | 1·5 | 5·1 (+) |
| | 4 | 2·4 | 2·8 (−) |
| Sub-cutaneous Ehrlich carcinoma in BALB/c (minus) mice | 1 | 3·9 | 3·4 (−) |
| | 2 | 4·9 | 5·6 (+) |
| | 3 | 3·0 | 4·0 (−) |
| | 4 | 2·9 | 2·4 (−) |
| | 5 | 7·5 | 7·2 (−) |
| | 6 | 2·7 | 4·3 (+) |
| | 7 | 3·2 | 2·9 (−) |

TABLE II.—*Dale–Schultz Tests for Tumour Antigen*

The response of the ileum to tumour serum 0·2% after desensitization to normal serum 0·2%. A result is rated positive (+) if more than 10% of maximum.

| Tumour type | Guinea-pig number | Dale-Schultz response (as % of maximum) | |
|---|---|---|---|
| Walker tumour in albino rats | 1 | 34 | (+) |
| | 2 | 47 | (+) |
| | 3 | 34 | (+) |
| | 4 | 22 | (+) |
| | 5 | 43 | (+) |
| Ehrlich ascites in BALB/c (minus) mice | 1 | 21 | (+) |
| | 2 | 12, 28 | (+) |
| | 3 | 27 | (+) |
| | 4 | 23 | (+) |

*Histamine-release tests.*—With Walker tumour in rats, in 2 of 3 animals tested the histamine-release in the tumour serum samples was significantly greater than that in the control samples at the level $P = 0{\cdot}05$. With the subcutaneous

Ehrlich carcinoma, a significantly increased histamine-release with tumour serum was obtained in 2 out of 6 animals.

*Dale–Schultz tests.*—Five out of 5 animals in the rat Walker tumour experiment and 4 out of 4 in the Ehrlich ascites experiment gave positive results.

Note that the results obtained with the two different tests are not strictly comparable as they were done on different animals and, in the case of the Ehrlich carcinoma, different forms of the tumour as well.

In the second part of the study the question posed was : " When positive results are obtained, are they specific for tumour serum? " These experiments were carried out with strain XXIIa hepatoma in inbred C3HA mice, a transplantable hepatoma in August rats and the Ehrlich carcinoma in inbred BALB/c (minus) mice. The results obtained with tumour serum were compared with those obtained with serum from animals with non-malignant proliferative lesions—pneumoderma pouches or regenerating liver. The results are given in Tables III and IV. The same guinea-pigs were used for both tests.

TABLE III.—*Histamine-Release Test for Tumour Antigen*

Results with various test sera (0·1%) after partial desensitization to normal serum (0·1%). Figures given are means of 3–4 samples. " NS " = normal serum. " CA.S " = tumour serum. " Prolif. S " = serum from animals with ⅔ hepatectomy. " Infl. S " = serum from animals with pneumoderma pouches. A result is rated as positive (+) if the difference between test samples and control samples is significant at 5% level on a *t*-test.

| Tumour used for sensitization | Guinea-pig number | Histamine-release (as % total histamine) in: Control samples with: NS | Test samples with: CA. S | Prolif. S | Infl. S |
|---|---|---|---|---|---|
| August rat hepatoma | 1 | 0·6 | 0·6 (−) | 1·1 (−) | 1·1 (−) |
| | 2 | 2·6 | 2·7 (−) | 2·8 (−) | 3·0 (−) |
| | 3 | 3·3 | 1·7 (−) | 1·7 (−) | 1·9 (−) |
| | 4 | 1·3 | 1·2 (−) | 1·2 (−) | 1·6 (+) |
| Strain XXIIa hepatoma in C3HA mice | 1 | 4·6 | 5·1 (−) | 4·7 (−) | 4·7 (−) |
| | 2 | 2·4 | 2·1 (−) | 2·5 (−) | 2·7 (−) |
| | 3 | 6·8 | 7·1 (−) | 7·5 (−) | 7·0 (−) |
| | 4 | 15·4 | 19·1 (+) | 16·7 (−) | 18·5 (−) |
| | 5 | 8·4 | 3·4 (−) | 3·5 (−) | 3·3 (−) |
| | 6 | 2·1 | 2·2 (−) | 1·9 (−) | 2·1 (−) |
| Ehrlich ascites in BALB/c (minus) mice | 1 | 1·7 | 2·7 (+) | 1·5 (−) | 2·2 (+) |
| | 2 | 3·6 | 5·1 (+) | 4·2 (−) | 6·9 (+) |
| | 3 | 4·2 | 4·4 (−) | 4·2 (−) | 4·4 (−) |
| | 4 | 2·3 | 2·7 (−) | 2·0 (−) | 2·5 (−) |
| | 5 | 0·9 | 1·5 (+) | 0·9 (−) | 0·7 (−) |

*Histamine-release tests.*—With the August rat hepatoma no positive results were obtained with either the tumour serum or the serum from animals with partial hepatectomy. One out of 4 animals with pneumoderma pouches gave a positive result. With the strain XXIIa hepatoma in C3HA mice, the tumour serum gave a positive result in 1 animal out of 6 and no positive results were obtained with serum from animals with pneumoderma pouches or partial hepatectomies. With the Ehrlich ascites tumour 3 out of 5 animals gave positive

TABLE IV.—*Dale–Schultz Test for Tumour Antigen*

Anaphylactic contractions of ileum with various test sera after prior desensitization to normal serum (0·2%). A response is rated as positive (+) if it is more than 10% of maximum. " Ca. S " = tumour serum. " Prolif. S " = serum from partially hepatectomized animals. " Infl. S " = serum from animals with pneumoderma pouches.

| Tumour used | Guinea-pig number | Dale-Schultz response as % of max. with: CA. S (0·2%) | Prolif. S (0·2%) | Infl. S (0·2%) |
|---|---|---|---|---|
| August rat hepatoma | 1 | 4 (−) | 2 (−) | 8 (−) |
| | 2 | 0 (−) | 0 (−) | 0 (−) |
| | 3 | 0 (−) | 0 (−) | 0 (−) |
| | 4 | 0 (−) | 0 (−) | 0 (−) |
| Strain XXIIa hepatoma in C3HA mice | 1 | 0 (−) | 0 (−) | 0 (−) |
| | 2 | 0 (−) | 0 (−) | 0 (−) |
| | 3 | 0 (−), 5 (−) | 0 (−) | 3 (−) |
| | 4 | 2 (−) | 0 (−) | 0 (−) |
| | 5 | 3 (−) | 0 (−) | 0 (−) |
| | 6 | 6 (−), 8 (−) | 0 (−) | 0 (−) |
| Ehrlich ascites in BALB/c (minus) mice | 1 | 18 (+) | 0 (−) | 8 (−) |
| | 2 | 6 (−), 14 (+) | 0 (−) | 23 (+), 8 (−) |
| | 3 | 0 (−) | 0 (−) | 12 (+), 28 (+) |
| | 4 | (no results obtainable, ileum hyperactive) | | |
| | 5 | (no results obtainable, ileum poorly sensitized) | | |

results with tumour serum, and 2 out of 5 gave positive results with serum from animals with pneumoderma pouches. No positives were obtained with the serum of animals with non-malignant proliferation of liver tissue.

*Dale–Schultz tests.*—With the August rat hepatoma and strain hepatoma in C3HA mice no positive results were obtained with any of the test sera. With the Ehrlich ascites in BALB/c (minus) mice 2 tests out of 4 with the tumour serum, and 3 tests out of 5 with " inflammation serum " gave positive results. (Note that one animal gave both positive and negative results with the same test sera in different experiments.) Serum from animals with partial hepatectomies did not give any positive results.

### DISCUSSION

The results of these experiments indicate that it is possible to obtain positive results with some tumours but not with others. The positive results are not, however, specific for tumour serum since similar positive results were obtained in several instances with the serum of animals with subacute inflammatory lesions.

Of the tumours used—the Walker carcinoma in albino rats, the Ehrlich carcinoma in inbred BALB/c (minus) mice, a transplantable hepatoma in August rats, and strain XXIIa hepatoma in inbred C3HA mice—in fact only the latter two were entirely satisfactory for the purpose. Only these two were tumours of fairly recent origin, developed in and passaged in the same inbred strain of mice, and could therefore be expected to be genetically identical with their hosts, (and even in the case of these tumours there may have been some genetic drift in the tumour or in the host strain). With the other two tumours (the Walker and

Ehrlich tumours) some degree of genetic difference between the tumour and the host was to be expected. It was felt, however, that even these far from ideal tumours provided better material for test in the first instance than clinical material.

Positive results with tumour serum were obtained mainly with these allogeneic tumours. In the case of the Ehrlich ascites tumour in mice, 3 out of 5 histamine tests and 6 out of 8 Dale–Schultz test were positive. This was perhaps not surprising—other immunological techniques have also given positive results with the Ehrlich mouse ascites. Wissler *et al.* (1956) reported that rabbit antiserum against this tumour inhibited the growth of the ascitic form of the tumour in other mice. (It is noteworthy that the antiserum was not effective against the subcutaneous form of the tumours.) Easty and Ambrose (1957) using Ehrlich tumour cell suspension and rabbit antisera in gel-diffusion and cytotoxic tests found that the Ehrlich ascites tumour cells contained soluble diffusible antigens absent from the normal kidney, spleen, liver and blood of the host strain of mice. (These authors did not however claim that the antigens were tumour-specific.) Takeda (1963) reported that rabbit antiserum against the Ehrlich carcinoma when absorbed with normal mouse powder still had an inhibitory effect on anaerobic glycolysis of tumour cells, but after absorption with packed tumour cells, this inhibitory effect disappeared. Bonmassar and Mariani (1962) reported positive cytotoxic and agglutinating tests with the Ehrlich ascites tumour.

Even if non-specific positive results had not been obtained in the present study, however, the results and those quoted above could not have been interpreted as necessarily indicating the presence of tumour-specific antigens because the tumour tissue and the normal tissue were not genetically identical.

The high proportion of positive results in the tests done with the Walker tumour was also not entirely surprising. The Walker tumour was grown in random-bred albino rats and here again there were genetic differences between tumour and host. Positive results obtained with tumour serum after desensitization to normal serum could be due to these differences. Another possible explanation is that there is an increase in concentration of a normal serum constituent. Darcy (1955, 1960) using gel-diffusion techniques, found high concentrations of $\alpha$-globulin in the serum of rats with Walker tumours. The $\alpha$-globulin was present in normal serum but was increased 15-fold in tumour growth. An increase in concentration of a normal serum constituent of this magnitude could also account for the positive results obtained with anaphylactic tests in the present study because, as was pointed out in a previous paper (Dale, 1965*b*), if one of the main antigens of the " desensitizing " solution is present in increased concentration in the challenging solution, a further positive response to that antigen can be obtained. Darcy found that the $\alpha$-globulin was also present in the serum of rats with actively growing tissue, (regenerating liver, kidney or skin) and in pregnant females. These finds were confirmed by Campbell, Kernot and Roitt (1959). In clinical studies employing physico-chemical methods non-specific changes in mucoproteins in neoplastic infections and other conditions have been described (Winzler, 1953 ; Lockey, Anderson and Maclagan, 1956) and it may be possible that something of the same sort occurred here. The positive results obtained in the present study with serum from animals with pneumoderma pouches may be another example of the same non-specific serum change. Although if the positive results with tumour serum and " inflammation serum " are due to a phenomenon similar to that described by Darcy, and by Campbell *et al.* it is surprising that the sera from animals with partial hepatectomies were consistently negative in all three groups of animals in which it was tested.

The negative results obtained with the mouse hepatoma were of particular interest as this was one experiment in which specific positive results might have provided suggestive evidence of the existence of tumour antigens. As it is, there was only 1 positive histamine-release test in 6 and no positive Dale–Schultz tests. This is all the more surprising when the findings of Abelev *et al.* (1963) with this same tumour are considered. Using gel-diffusion and immuno-electrophoresis these authors showed that an $\alpha$-globulin, not present in normal mouse liver or serum (though present in the embryonic mouse) was formed by the tumour and was present in the serum of tumour-bearing mice. This substance, though apparently readily detectable by double-diffusion in gel and immuno-electrophoresis was not detected by the supposedly more sensitive anaphylactic tests.

The results obtained in this study are at variance with those obtained with rabbit tumours by Shevliaguin (1959) who, using guinea-pigs sensitized with Brown–Pearce carcinoma extracts obtained positive anaphylactic responses with the serum from rabbits with Brown–Pearce carcinomata and negative results with the serum of rabbits with non-malignant conditions such as Shope papillomata, acute inflammation with necrosis, etc. Shevliaguin did not, however, interpret his results as necessarily indicating the presence of a specific tumour antigen. In fact in a subsequent study he reported positive results with tumour serum in guinea-pigs sensitized with *normal* tissue extracts (1961).

### SUMMARY

Anaphylactic tests for tumour antigen gave positive results in some animal tumours, but the results were not specific for neoplasia.

I am indebted to Professor H. O. Schild and Dr. J. L. Mongar for valuable discussion and advice. This work was supported by a grant from the British Empire Cancer Campaign for Research.

### REFERENCES

Abelev, G. I., Perova, S. D., Kramkova, N. I., Postnikova, Z. A. and Irlin, I. S.—(1963) *Transplantation*, **1**, 174.
Artamonova, V. A.—(1959) ‘Pathogenesis and Immunology of Tumours’. Edited by Vygodchikov, G. V., London (Pergamon Press) p. 49.
Bonmassar, E. and Mariani, L.—(1962) *Archo ital. Patol. clin. Tum.*, **5**, 415.
Burrows, D.—(1958) *Br. med. J.*, i, 368.
Campbell, P. N., Kernot, B. A. and Roitt, I. M.—(1959) *Biochem. J.*, **71**, 155.
Dale, M. M.—(1965*a*) *Immunology*, **8**, 435.—(1965*b*) *Immunology*, **8**, 444.—(1965*c*) *Br. J. Cancer*, **19**, 613.
Darcy, D. A.—(1955) *Nature, Lond.*, **176**, 225.—(1960) *Br. J. Cancer*, **14**, 534.
Easty, G. C. and Ambrose, E. J.—(1957) *Br. J. Cancer*, **11**, 287.
Gorodilova, V. V. and Shershul’skaia, L. V.—(1959) ‘Pathogenesis and Immunology of Tumours’. Edited by Vygodchikov, G. V., London (Pergamon Press) p. 101.
Hackett, E. and Gardonyi, E.—(1960) *Br. med. J.*, i, 1785.
Higgins, G. M. and Anderson, R. M.—(1931) *Archs Path.*, **12**, 186.
Korosteleva, T. A.—(1959) ‘The Pathogenesis and Immunology of Tumours’. Edited by Vygodchikov, G. V., London (Pergamon Press) p. 142.

LEVINA, D. M.—(1959) ' The Pathogenesis and Immunology of Tumours '. Edited by Vygodchikov, G. V., London (Pergamon Press) p. 113.
LOCKEY, E., ANDERSON, N. J. AND MACLAGAN, N. F.—(1956) *Br. J. Cancer,* **10**, 209.
MAAS, H. AND SCHNIEWIND, H.—(1960) *Klin. Wschr.*, **38**, 1164.
MAKARI, J.—(1955) *Br. med. J.*, ii, 1291.
MCEWEN, L. M.—(1959) *Br. med. J.*, ii, 615.
MAVER, M. E. AND BARRETT, M. K.—(1943) *J. natn. Cancer Inst.*, **4**, 65.
MONGAR, J. L. AND SCHILD, H. O.—(1960) *J. Physiol., Lond.*, **150**, 546.
RYZEWSKA, A.—(1962) *Nowotwory,* **12**, 85.
SELYE, H.—(1953) *J. Am. med. Ass.*, **152**, 1207.
SHEVLIAGUIN, V. I.—(1959) *Vop. Onkol.*, **5**, 149.—(1961) *Ibid.*, **7**, 57.
TAKEDA, K.—(1963) *J. Okayama med. Ass.*, **75**, 91.
WEINBREN, K.—(1959) *Gastroenterology,* **37**, 657.
WINZLER, R. J.—(1953) *Adv. Cancer Res.*, **1**, 506.
WISSLER, R. W., BARKER, P. A., FLAX, H. M., LAVIA, M. F. AND TALMAGE, D. W.—(1956) *Cancer Res.*, **16**, 761.
WITTIG, G., TEICHMANN, B. AND SCHNEEWEISS, U.—(1962) *Acta biol. med. germ.*, **8**, 274.
ZILBER. L. A.—(1958) *Adv. Cancer Res.*, **5**, 291.